# Inhibition of Mettl3-mediated m6A RNA modification of HMGCS1 protects retinal ganglion cells from glutamate excitotoxicity-induced ferroptosis in a rat model of glaucoma

**DOI:** 10.1097/JS9.0000000000003213

**Published:** 2025-08-20

**Authors:** Chao Wang, Lemeng Feng, Weizhou Fang, Cheng Zhang, Wulong Zhang, Weiming Zhu, Ye He, Zhaohua Xia, Weitao Song, Xiaobo Xia

**Affiliations:** a Eye Center of Xiangya Hospital, Central South University, Changsha, Hunan, P.R. China; b Hunan Key Laboratory of Ophthalmology, Changsha, Hunan, P.R. China; c National Clinical Key Specialty of Ophthalmology, Changsha, Hunan, P.R. China; d National Clinical Research Center for Geriatric Diseases (Xiangya Hospital), Central South University, Changsha, Hunan, P.R. China

**Keywords:** ferroptosis, glaucoma, HMGCS1, m6A RNA modification, Mettl3, retina ganglion cell

## Abstract

**Background:**

Glaucoma, a leading cause of irreversible blindness worldwide, is characterized by progressive retinal ganglion cells (RGCs) degeneration. The accumulation of glutamate in the retina is a common mechanism underlying the of RGCs death in various forms of glaucoma. Neuroprotective strategies for RGCs remain an unmet need, urging exploration of novel molecular mechanisms beyond intraocular pressure (IOP) control.

**Materials and methods:**

Retinal m6A methylation sequencing, RT-qPCR, and dot blot techniques were performed to identify m6A methylation levels change in an N-methyl-D-aspartate (NMDA)-induced glaucoma model of rats and find potential targets. The effect of methyltransferase-like 3 (Mettl3) inhibition on ferroptosis of R28 and RGCs glutamate excitotoxicity model was detected by using Mettl3 inhibitor STM2457, Cell Counting Kit-8 (CCK-8), FerroOrange, Liperfluo, malondialdehyde (MDA) assay, glutathione (GSH) assay, immunofluorescence staining, Hematoxylin-Eosin (H&E) staining and flash visual evoked potential (f-VEP) were used to study the ferroptosis in R28 and RGCs. Using si-RNA and oe-RNA to knockdown YTHN6-methyladenosine RNA binding protein 2 (YTHDF2) or Hydroxyl-3-methylglutaryl-Coenzyme A synthase 1 (HMGCS1) in the R28. Lentiviral vectors were applied to overexpress the HMGCS1 in rat retina. MeRIP-qPCR and Western Blot to study the mechanism of how Mettl3 regulates m6A methylation and expression of downstream targets.

**Results:**

Our findings demonstrate that NMDA-induced excitotoxicity significantly elevated retinal m6A methylation levels. HMGCS1 m6A methylation was significantly increased while its expression significantly decreased in the NMDA group. In R28 cells, inhibition of Mettl3 significantly alleviated glutamate excitotoxicity-induced R28 and RGCs ferroptosis and restored the visual function of rats. Knockdown HMGCS1 significantly reduced the protective effect of Mettl3 inhibition on the R28 cells and overexpress HMGCS1 protected R28 cell and RGCs from NMDA-induced glutamate excitotoxicity. YTHDF2 reverses this protective effect by recognizing and degrading m6A-modified HMGCS1 mRNA, thereby promoting ferroptosis.

**Conclusion:**

Our study investigates the inhibition of Mettl3-mediated m6A RNA modification of HMGCS1 as a critical regulator of RGC ferroptosis in glaucoma, providing a potential therapeutic target for glaucoma.

## Introduction

Glaucoma is the leading cause of irreversible blindness worldwide, characterized by visual field defects and vision loss due to the selective and progressive death of retinal ganglion cells (RGCs)^[1]^. By 2040, glaucoma is projected to impact 111.8 million people worldwide^[2]^. The projected number of glaucoma patients in Europe is expected to reach 13.63 million by 2045^[3]^. Currently, there are no effective clinical therapies to repair or reverse damage to RGCs. Elevated intraocular pressure (IOP) is recognized as one of the most significant risk factors for glaucoma. Lowering IOP is currently the only effective method to control the progression of glaucoma in clinical practice^[4]^. However, some patients develop glaucoma with typical visual field defects and optic nerve damage without experiencing elevated IOP^[5,6]^. Additionally, some glaucoma patients who initially presented with elevated IOP continue to experience progressive visual field loss despite well-controlled IOP after treatment^[5,6]^. This indicates that elevated IOP is not the sole factor causing RGCs damage in glaucoma. Our previous studies indicate that the mechanisms of RGCs death in glaucoma are highly complex, involving multiple forms of cell death, including apoptosis^[7]^, pyroptosis^[7,8]^, necroptosis^[9]^, ferroptosis^[10,11]^, and PANoptosis, a complex form of cell demise is characterized by crosstalk and coordination among pyroptosis, apoptosis, and necroptosis^[12]^.

Glutamate is the primary excitatory neurotransmitter in the central nervous system. Under physiological conditions, glutamate acts as an excitatory neurotransmitter by binding to N-methyl-D-aspartate (NMDA) receptors, participating in RGCs signal transduction^[13,14]^. However, in the pathological state of glaucoma, excessive glutamate in retinal tissue can cause overactivation of NMDA receptors on RGCs, leading to calcium influx. This calcium overload activates multiple cell death pathways, including calpain activation, oxidative stress, and endoplasmic reticulum stress, resulting in irreversible damage to RGCs^[15–17]^. As a classical inducer of ferroptosis, glutamate can induce ferroptosis, a novel form of cell death distinct from other types in terms of morphology, biology, and genetics, characterized by iron-dependent lipid peroxidation^[18]^. Numerous diseases, including tumors, neurodegenerative diseases^[19,20]^. In glaucoma, including acute ocular hypertension model,^[10,21,22]^ optic nerve crush injury model^[23]^, and NMDA-induced glutamate excitotoxicity^[11]^, ferroptosis has also been found to play a significant role. These studies suggest that ferroptosis is a potential therapeutic target for glaucoma.

RNA methylation, as an epigenetic modification, affects gene expression at the post-transcriptional level. m6A RNA methylation is one of the most common and abundant methylation modifications in eukaryotic mRNA. As a reversible mRNA modification, the methylation level of m6A in cells is regulated by the interplay between methyltransferases (writers) and demethylases (erasers) and is subsequently recognized directly or indirectly by m6A-binding proteins (readers). This dynamic regulation can influence gene expression, playing crucial roles in physiological and pathological processes. The m6A RNA modification primarily occurs in conserved RRACH motifs (R = A or G, H = A, C, or U), enriched in stop codons, 3ʹ untranslated regions (3ʹ UTRs), and long internal exons, with its levels dynamically regulated by cell states^[24]^. m6A RNA methylation alterations are significantly associated with various neurodegenerative diseases, such as Alzheimer’s disease^[25]^, Parkinson’s disease^[26]^, and multiple sclerosis^[27]^. However, research on its involvement in glaucoma is still quite limited.

Hydroxyl-3-methylglutaryl-Coenzyme A synthase 1 (HMGCS1) is a key enzyme involved in the early stages of cholesterol biosynthesis. It catalyzes the condensation of acetyl-CoA and acetoacetyl-CoA to form 3-hydroxy-3-methylglutaryl-CoA (HMG-CoA), a crucial intermediate in the synthesis of cholesterol and other isoprenoids. HMGCS1, encoded by the HMGCS1 gene on human chromosome 5q13.3, plays a vital role in maintaining cellular cholesterol homeostasis, membrane fluidity, and the synthesis of steroid hormones and bile acids. Disruption of HMGCS1 activity can lead to cholesterol metabolism disorders, resulting in hypercholesterolemia, atherosclerosis, and metabolic imbalances^[28]^. Some studies suggest a potential link between HMGCS1 and the pathogenesis of glaucoma^[29,30]^, as well as its connection to ferroptosis^[28,31]^. Our previous gene screening also revealed the potential role of HMGCS1 in glaucoma and ferroptosis, highlighting the importance of understanding HMGCS1’s regulatory mechanisms and functional significance for developing novel therapeutic strategies for glaucoma.

**HIGHLIGHTS**

- Glutamate excitotoxicity induces upregulation of m6A RNA modification level in the retina of a glaucoma model of rats.
- The glutamate excitotoxicity-driven Mettl3/HMGCS1 axis facilitates ferroptosis in retinal ganglion cells.
- M6A methylation of HMGCS1 induced by glutamate excitotoxicity is recognized by YTHDF2.

The model of glutamate excitotoxicity induced RGCs injury is widely used to simulate the pathological state of RGCs in glaucoma in both *in vitro* and *in vivo* studies. Research has shown significant increases in retinal glutamate levels across various types of glaucoma, making RGCs excitotoxicity a common characteristic of RGCs injury.

This article is compliant with the TITAN Guidelines 2025^[32]^ In this study, we aim to explore the injury and characteristic changes of RGCs induced by glutamate excitotoxicity and investigate the role of m6A RNA methylation, providing evidence that intervention and regulation at the m6A RNA methylation level hold potential therapeutic significance. This insight bridge the knowledge gap by exploring how Mettl3-mediated m6A modifications regulate HMGCS1 expression and ferroptosis in retinal ganglion cells during glaucomatous injury, a mechanism not previously elucidated in the context of neurodegeneration.

## Materials and methods

### NMDA-induced glaucoma model of rats

The research project listed below has been reviewed and approved by the Institutional Animal Care and Use Committee (IACUC) of Central South University (approval number: CSU-2023-0297). Experiments were carried out on SD male rat aged 7 weeks, weighted from 250 to 260 g. The rats were obtained from Hunan SJA Laboratory Animal Co., Ltd (Changsha, Hunan, China) [license number: SYXK (Xiang) 2020-0019]. Before experimentation, the animals were adaptively fed for one week under Specific pathogen Free (SPF) feeding conditions of a 12 h cycle of light and dark at a temperature of 21 ± 1°C. The standard diet was purchased from Hunan SJA Laboratory Animal Co., Ltd (Changsha, Hunan, China). Its main ingredients include corn, wheat, fish meal, vegetable oil, and vitamin B6. It is suitable for long-term maintenance and various experimental needs, and it is compatible with high-pressure sterilization. Food and water were available *ad libitum*. All the experiments were performed in accordance with the Association for Research in Vision and Ophthalmology (ARVO) Statement for the Use of Animals in Ophthalmic and Vision Research. This work has been reported in accordance with the ARRIVE guidelines^[33]^.

We randomly divided the rats into three groups (5 rats per group): NMDA group (20 mM), NMDA + STM2457 group (20 mM NMDA + 1 mM STM2457), NMDA + FB23 group (20 mM NMDA + 1 mM FB23), NMDA + oe-HMGCS1group and control group (sterile saline). STM2457 (T9060) was from Topscience (Shanghai, China). NMDA (M3262) was from Sigma-Aldrich (St. Louis, MO, USA). All the rats were anesthetized with pentobarbital (1%, 80 mg/kg, intraperitoneal injection). Used oxybrucaine hydrochloride (Shentian Pharmaceutical Co., Ltd.) eye drop for ocular surface anesthesia and tropicamide phenylephrine (Shentian Pharmaceutical Co., Ltd.) eye drop for pupil dilation. A 34 G needle was inserted into the vitreous cavity at the limbus. 4 µL of drug fluid was injected into the vitreous cavity through the above incision using a microsyringe (Hamilton, Reno, NV, USA). The control group received intravitreal injection with the same volume of saline. The entire operation is performed under a stereomicroscope.

### Intravitreal lentivirus administration

As described earlier, rats were randomly grouped, and animals in the NMDA + oe-HMGCS1 group were subsequently administered an intravitreal lentivirus. Lentivirus was purchased from GenePharma (Suzhou, China). SD male rats were anesthetized with pentobarbital (1%, 80 mg/kg, intraperitoneal injection). Ocular surface anesthesia was achieved with oxybuprocaine hydrochloride eye drops (Shentian Pharmaceutical Co., Ltd.), and pupil dilation was achieved using tropicamide phenylephrine eye drops (Shentian Pharmaceutical Co., Ltd.). A 34-gauge needle was inserted into the vitreous cavity at the limbus, and the lentivirus was injected 1 mm posterior to the limbus into the vitreous cavity. Lentivirus carrying the GFP fluorescent tag (LV-HMGCS1) was injected into the eye. One day later, the NMDA model was established and drug treatments were administered.

### Immunofluorescence staining of retinal whole-mounts

Rats were sacrificed 3 days after intravitreal injection. Fixed the eyeballs in 4% paraformaldehyde for 30 min, and then dissected the retinas under a stereomicroscope. Blocked the retina into 2% Bovine Serum Albumin (BSA) (Servicebio, Wuhan, China) prepared in phosphate buffer saline (PBS) containing 0.5% Triton-X100 (Servicebio, Wuhan, China) for 1 h at room temperature. After the addition of primary anti-Brn3a antibody (abcam; Cambridge; #ab245230; 1:100), the retina was put in a refrigerator overnight at 4°C. Then washed the retina with 0.5% Triton-X10 3 times. Then fixed the retina in 4% paraformaldehyde for 10 min and washed it. The retina was incubated with the Goat Anti-Rabbit IgG H&L, Alexa Fluor 488 (abcam; Cambridge; 1:200) for 1.5 h at room temperature and washed with PBS. Photographed the retina using a fluorescence microscope. The immunoreactivity and RGCs number of the sections was analyzed by ImageJ software.

### m6A dot blot assay

We collected retina samples from two groups SD male rat. Total RNA was isolated and purified using TRIzol reagent (Invitrogen, Carlsbad, CA, USA) following the manufacturer’s procedure. The RNA amount and purity of each sample was quantified using NanoDrop ND-1000 (NanoDrop, Wilmington, DE, USA). The RNA samples were subsequently diluted to a final concentration of 200/100/50 ng/μL and incubated at 95°C for 3 minutes. The samples were then immediately cooled on ice to prevent reformation of RNA secondary structures. Positive charge Nylon membranes (Biosharp, Beijing, China) were cut to appropriate sizes. Circular marks were left on the nylon membrane using the blunt end of a 1 ml pipette tip to guide sample application. The membrane was transferred to a clean 10 cm culture dish. 2 μL of RNA was spotted onto the membrane. The nylon membrane was transferred to a 37°C oven for RNA-membrane crosslinking for 30 minutes. Then washed membrane in 10 mL TBST (1X TBS, 0.1% Tween-20) for 5 minutes to remove unbound RNA. The membrane was incubated with blocking solution at room temperature for 1 hour. Discarding the blocking solution, the membrane was incubated at 4°C overnight with the m6A antibody (#202003; Synaptic Systems; Goettingen, Germany). The membrane was washed three times with TBST for 10 minutes each at room temperature. The membrane was then incubated with the secondary antibody at room temperature for 1 hour. Then washed the membrane three times with TBST for 10 minutes each. Following a 5-minute incubation with ECL substrate. Subsequently, the membrane was transferred to a solution containing methylene blue staining buffer (0.2 M sodium acetate and 0.4% methylene blue in 0.4 M acetic acid) at room temperature for 30 minutes. The membrane was briefly rinsed with ddH2O for 60 seconds. The methylene blue-stained membrane was imaged using white light.

### RNA extraction and fragmentation

Total RNA was extracted as explained above. The RNA integrity was assessed by Bioanalyzer 2100 (Agilent, CA, USA) with RIN number >7.0, and confirmed by electrophoresis with denaturing agarose gel. Poly (A) RNA is purified from 50 μg total RNA using Dynabeads Oligo (dT)25-61005 (Thermo Fisher, CA, USA) using two rounds of purification. Then the poly(A) RNA was fragmented into small pieces using Magnesium RNA Fragmentation Module (NEB, cat. e6150, USA) under 86°C for 7 min.

### m6A immunoprecipitation and library construction

The cleaved RNA fragments were incubated for 2 h at 4°C with m6A-specific antibody (# 202003; Synaptic Systems, Germany) in IP buffer (50 mM Tris-HCl, 750 mM NaCl and 0.5% Igepal CA-630). Then the IP RNA was reverse-transcribed to create the cDNA by SuperScriptTM II Reverse Transcriptase (#1896649; Invitrogen, USA), which were next used to synthesize U-labeled second-stranded DNAs with E. coli DNA polymerase I (#m0209; NEB, USA), RNase H (#m0297; NEB, USA) and dUTP Solution (#R0133; Thermo Fisher, USA). An A-base is then added to the blunt ends of each strand, preparing them for ligation to the indexed adapters. Each adapter contains a T-base overhang for ligating the adapter to the A-tailed fragmented DNA. Single- or dual-index adapters are ligated to the fragments, and size selection was performed with AMPureXP beads. After the heat-labile UDG enzyme (#m0280; NEB, USA) treatment of the U-labeled second-stranded DNAs, the ligated products are amplified with PCR by the following conditions: initial denaturation at 95°C for 3 min; 8 cycles of denaturation at 98°C for 15 sec, annealing at 60°C for 15 sec, and extension at 72°C for 30 sec; and then final extension at 72°C for 5 min. The average insert size for the final cDNA library was 300 ± 50 bp. At last, we performed the 2 × 150 bp paired-end sequencing (PE150) on an illumina NovaseqTM 6000 (LC-Bio Technology CO., Ltd., Hangzhou, China) following the vendor’s recommended protocol.

### Bioinformatics analysis of m6A-seq and RNA-seq data

The fastp software (https://github.com/OpenGene/fastp) was used to remove the reads that contained adaptor contamination, low quality bases and undetermined bases with default parameter. Then sequence quality of IP and Input samples were also verified using fastp. We used HISAT2 (http://daehwankimlab.github.io/hisat2) to map reads to the reference genome of rat. For m6A peak identification, aligned reads from IP and Input libraries were analyzed using the exomePeak R package (https://bioconductor.org/packages/exomePeak). This package performs m6A peak calling based on enrichment signals in the IP samples compared to Input controls and outputs results in BED or BigWig formats suitable for visualization in IGV (http://www.igv.org). To investigate m6A binding motifs, we performed de novo and known motif analysis using MEME (http://meme-suite.org) and HOMER (http://homer.ucsd.edu/homer/motif). Motif distribution relative to peak summits was also evaluated. The genomic features of identified m6A peaks were annotated using ChIPseeker (https://bioconductor.org/packages/ChIPseeker) by intersecting peak regions with gene architecture. Then StringTie (https://ccb.jhu.edu/software/stringtie) was used to perform expression level for all mRNAs from Input libraries by calculating FPKM (total exon fragments /mapped reads (millions) × exon length (kB)). The differentially expressed mRNAs were selected with log2 (fold change) > 1 or log2 (fold change) <−1 and *P* value <0.05 by R package edgeR (https://bioconductor.org/packages/edgeR).

### Cell culture and glutamate excitotoxicity-induced ferroptosis model of R28 cells

Rat retinal precursor (R28) cells (Cat# CVCL_5I35) were provided by Central South University (Changsha, China). In our previous experiments, we found that the isolation and culture of primary RGCs were challenging, and their growth status often deteriorated after passaging. Therefore, referring to several high-quality international studies, we utilized the widely recognized r28 cell line to establish an *in vitro* experimental model^[9,34–36]^. R28 cells were maintained with Dulbecco’s modified Eagle’s medium (DMEM; Procell, Wuhan, China) containing 1 g/L glucose and supplemented with 10% FBS (ExCell Bio, Shanghai, China) at 37°C with 5% CO2. Glutamate was dissolved in cell culture medium to a concentration of 10 mM. Added the glutamate and incubated the cells in the condition of 37°C and 5% CO_2_ for 24 hours.

### Gene modulation in R28 cells

For targeted gene silencing, R28 cells were transfected with 50 nM of specific small interfering RNAs (siRNAs) against either HMGCS1 (si-HMGCS1; Gene Pharma, Suzhou, China) or YTHN6-methyladenosine RNA binding protein 2 (YTHDF2) (si-YTHDF2; Gene Pharma, Suzhou, China) using Lipofectamine 3000 transfection reagent (Gene Pharma, Suzhou, China). For HMGCS1 overexpression, cells were transfected with 2 μg of LV-HMGCS1 overexpression plasmid (oe-HMGCS1; Gene Pharma, Suzhou, China). Transfection efficiency was validated 48 hours post-transfection through qRT-PCR or Western blot.

### CCK-8 assay

Cell viability was analyzed using Cell Counting Kit-8 (Topscience, Shanghai, China) according to the manufacturer protocols. Seeded R28 into 96-well culture plates (5 × 10^3^ cells/well). Then divided the cells into several groups and treated with different drugs. After treatment for 24 hours, removed the culture medium and added the culture medium containing 10% CCK-8 solution and then cultured for 2 hours at 37 °C. The absorbance was analyzed at 450 nm with a Synergy LX multi-detection microplate reader. The cell viability was calculated (n = 3): relative cell viability (%) = (absorbance at 450 nm of treated group − absorbance at 450 nm of blank)/(absorbance at 450 nm of control group − absorbance at 450 nm of blank) × 100.

### Calcein AM/PI cell viability assay

R28 cells were seeded and cultured at a density of 10 × 10^4^/well in 1 ml of medium into 12-well microplates. Divided the cells into several groups and treated for 24 h. Calcein AM/PI staining was performed after the cells were treated with different drugs for 24 hours using Calcein/PI Cell Viability Assay Kit (Beyotime; Shanghai, China). Observed the cells under a fluorescence microscope (n = 5).

### Transmission electron microscopy

R28 cells (2 × 10^5^ cells/well) seeded in a 6-well plate were cultured in DMEM and 10% FBS containing 10 mM glutamate and 25 μM STM2457 for 24 hours. The cells were fixed with 2.5% glutaraldehyde fixative (Servicebio; Wuhan, China) at room temperature in the dark for 5 minutes. The cells were scraped off with a cell scraper and centrifuged (100 × g, 5 min). The fixative was discarded and fresh electron microscope fixative was added (Servicebio; Wuhan, China). The cells were fixed at room temperature for 30 minutes in the dark. Next, 1% osmium tetroxide (Servicebio) was used for post-fixing at room temperature (20°C) for 2 hours. After dehydration with gradient concentrations of alcohol (50%, 70%, 80%, 90%, and 100%) and acetone, cells were embedded in epoxy resin (Servicebio). The samples were cut into 60–80 nm ultra-thin sections for uranium-lead (Servicebio) double staining and then examined under a transmission electron microscope (Hitachi, Tokyo, Japan).

### Detection of intracellular reactive oxygen species (ROS)

The intracellular ROS were detected with the ROS assay kit (Beyotime, Shanghai, China). After 24 hours of drug treatment, cells were collected by trypsinization. Stained the cells with 20 μM DCFDA medium and incubated at 37° C for 30 minutes. Then washed cells with wash buffer for 3 times and analyzed on flow cytometer immediately (n = 4). The DCF was excited by a 488 nm laser and the fluorescence intensity was detected at 535 nm. The flow cytometry results were analyzed with Flowjo software.

### Intracellular lipid peroxide and intracellular ferrous ions detection

R28 cells were seeded in a 48-well plate (2x10^4^ cells/well). After 24 hours of adaptive growth on 9 mm cell slides (Biosharp; Anhui, China). Randomly divided cells into different group (n = 5). Added the drug and incubated the cells in the condition of 37°C and 5% CO_2_ for 24 hours. Washed the cells twice with HBSS. Added 200 μl 1 μM Liperfluo HBSS solution (Dojindo Laboratories; L248; Tokyo, Japan). After incubating at 37°C in a 5% CO_2_ incubator for 30 min, then washed twice with HBSS. Liperfluo is selectively oxidized by lipid peroxides, and the oxidized form of Liperfluo exhibits strong fluorescence at the cell membrane (Ex: 524 nm and Em: 535 nm). For the intracellular ferrous ions detection, we added 1 μmol/l FerroOrange (Dojindo Laboratories; M374; Tokyo, Japan) working solution and incubated for 30 minutes. Then observed the fluorescence with a fluorescence microscope (Nikon; Tokyo, Japan). The brightness parameters were consistent between the groups during image capture.

### Malondialdehyde (MDA) detection

MDA content was detected with a malondialdehyde (MDA) content detection kit (#AKFA013M; Boxbio; Beijing, China). Randomly divided cells into different group (n = 3). Cell grouping and drug treatment were performed in the same way as the intracellular lipid peroxidation detection. Used the trypsinization to collect cells and added MDA extract. Then centrifuged (8000 xg) for 10 min at 4°C. The protein concentration was detected by BCA Protein Assay Kit (Beyotime; Shanghai, China). Added the MDA detection working solution into the sample, then treated the compound in boiling water bath for 60 min. After cooling to room temperature in an ice bath, centrifuge at 10 000 xg for 10 min at room temperature. Pipette 200 µL of the supernatant into a 96-well plate, measure the absorbance at 450 nm, 532 nm and 600 nm with a Synergy LX multi-detection microplate reader (BioTek; USA). Then calculate the MDA content with the formula: MDA content (nmol/mg prot) = (6.45 × (ΔA532 − ΔA600) 1.29 × ΔA450) × Vtotal ÷ (Cpr × Vsample) = 5 ×(6.45 ×(ΔA532 − A600) − 1.29 × ΔA450) ÷ Cpr.

### Intracellular glutathione (GSH) detection

Micro GSH assay kit (BC1175; Solarbio, Beijing, China) was used to detect the content of reduced GSH. Cell grouping and drug treatment were performed in the same way as the intracellular lipid peroxidation detection (n = 3). After 24 hours of drug treatment, collected 5000 cells using 0.25%Trypsin-EDTA (NCM Biotech; Wuhan, China). Washed the cells by PBS for 2 times. After adding GSH extract, used the liquid nitrogen and 37°C water bath to freeze and thaw the sample twice. Centrifuged (x 8000 g) at 4°C for 10 min. GSH content was detected according to the manufacturer’s instructions and normalized to cell number. GSH content (μg/10^6^cell) = x × V ÷ (Vsupernatant liquid volume ÷ Vtotal volume × N) = x ÷ N.

### Methylated RNA immunoprecipitation (MeRIP)

RNA was extracted as explained previously. The RNA was divided into two equal portions. One part was used for immunoprecipitation (IP), and the other part was kept as an input for use as a control. According to the manufacturer’s protocol, Dynabeads™ Antibody Coupling Kit (14311D; Invitrogen, California, United States) was pre-incubated with the m6A antibody (#202003; Synaptic Systems, Germany) to generate m6A-immunomagnetic beads. In the IgG group, the magnetic beads were incubated with rabbit serum-derived IgG (I8140; Sigma-Aldrich, MO, USA) to form the control complex. RNA was added to the m6A-immunomagnetic beads along with m6A binding buffer (10 mL: 0.5 mL Tris-HCl (pH 7.4, 1 M), 1.5 mL NaCl (5 M), 0.5 mL NP-40 (10% vol/vol stock), 10 μL Inhibitor, topped up with ddH_2_O to 10 mL). Once m6A-immunomagnetic beads were adequately bound to the RNA, Elution buffer (50 mL: 10 mL 0.1 M DTT, 0.44 g NaCl, 2.5 mL pH 7.5 1 M Tris-HCl, 0.1 mL 0.5 M EDTA, 0.5 mL 10% SDS, 10 μL Inhibitor, topped up with ddH_2_O to 50 mL) was used to elute the m6A RNA from the immunomagnetic column. RNA purification was performed using the microRNA purification kit (R6247-00; Omega, Norcross, United States). cDNA synthesis was carried out using a reverse transcription kit. Subsequently, qRT-PCR was performed (n = 3). Then calculate the fold change of m6A RNA with the formula:

ΔCt = Ct_IP_ − Ct_Input_

ΔΔCt = ΔCt treated group − ΔCt control group

Fold change (relative expression) = 2^(−ΔΔCt)^

### Western blotting assay

24 hours after drug treated, RIPA lysis buffer (Beyotime; Nanjing, China) was used to extract proteins from R28 cells. The protein concentration was quantified by using BCA Protein Assay Kit (Beyotime; Shanghai, China)). Loaded the protein at 12% SDS-PAGE and then transferred to PVDF membranes (Millipore; MA, USA). Then incubated the membrane with blocking buffer (5% nonfat milk in TBST) for 90 min at room temperature. Subsequently, then incubated membranes with primary antibodies overnight at 4°C: beta-actin monoclonal antibody (# 66009-1-Ig; Proteintech; 1:1000), SLC7A11 Polyclonal Antibody (#PA1-16893; Thermofisher; 1:1000), HMGCS1 Polyclonal antibody (#17643-1-AP; Proteintech; 1:1000), glutathione peroxidase 4 (GPX4) antibody (#67763-1-Ig; Proteintech; 1:1000), YTHDF2 Polyclonal antibody (#24744-1-AP; Proteintech; 1:1000). The next day, washed the membranes and incubated it in respective secondary antibodies for 90 min in room temperature. The bands signals were monitored by enhanced chemiluminescence reagent (Bio-Rad; USA). The grayscale intensities quantified with ImageJ software were normalized to the control and β-actin (n = 3).

### qRT-PCR

Total RNA was extracted from the R28 cells using TRIzol reagent (Invitrogen, Waltham, MA, USA), and cDNA was synthesized with a Hifair III 1st Strand cDNA Synthesis Kit (Yeasen Biotechnology; Shanghai, China). Quantitative real-time polymerase chain reaction (RT-PCR) was performed using a Hieff qPCR SYBR Green Master Mix (Low Rox; Yeasen Biotechnology; Shanghai, China) with a sequence detection system (Prism 7500; Applied Biosystems; Waltham, MA, USA) according to the manufacturer’s instructions. The specific primers were designed by Sangon Biotech (Shanghai, China), including HMGCS1 (forward 5′-CACAGCCGCAGTCTTCAATG-3′ and reverse 5′-GCGTTTCCTGAGGCATATATAGC-3′), YTHDF2 (forward 5′-ACAGGCAAGGCCGAATAATG-3′ and reverse 5′-GGCTGTGTCACCTCCAGTAG-3′), Mettl3 (forward 5′-GTGCATGAAAGCCAGTGACG-3′ and reverse 5′-CTTGCTGCCAGGACTCTCAG-3′), fat mass and obesity-associated protein (FTO) (forward 5′-GTCAGAGAGAAGGCCAATGAA-3′ and reverse 5′-CTCTGCTCTTAAGGTCCACTTC-3′) and β-Actin (forward 5′-CTACAATGAGCTGCGTGTGGC-3′ and reverse 5′-CAGGTCCAGACGCAGGATGGC-3′) Relative mRNA levels were normalized to those of β-actin and were calculated using the 2^−ΔΔCT^ method. Each sample was measured in triplicate wells, and the experiments were repeated three times.

### RNA stability assay

R28 cells were transfected with si-YTHDF2, and then treated with 5 μg/mL actinomycin D (HY-17,559; MedChemExpress) at 24-hours post-transfection for 0 h, 24 h, or 48 h. Then followed by RNA extraction. qRT-PCR was used to detect the relative expression of HMGCS1 mRNA at various time points (n = 3).

### Immunofluorescence (IF) staining of R28 cells

R28 cells from all groups were fixed in 4% paraformaldehyde for 15 min. Cells were then washed three times with PBS and permeabilized with 0.1% Trixton-X-100 in PBS for 10 min. After blocking with 5% BSA (m/v) for 30 min, cells were immunostained with SLC7A11 Polyclonal Antibody (#PA1-16893; Thermofisher; 1:200), HMGCS1 Polyclonal antibody (#17643-1-AP; Proteintech; 1:200), glutathione peroxidase 4 (GPX4) antibody (#67763-1-Ig; Proteintech; 1:200), YTHDF2 Polyclonal antibody (#24744-1-AP; Proteintech; 1:200) with 5% BSA(m/v) at 4 °C overnight. Following another three washes with PBS, cells were incubated with the appropriate secondary antibody of anti-rabbit IgG Alexa Fluor 594 (1:200; Beyotime), anti-mouse IgG Alexa Fluor 594 (1:200; Beyotime). Actin-Tracker Green-488 (1:200, Beyotime) with 5% BSA(m/v) for 2 h. Cells were then washed five times with PBS and stained with 4′,6-diamidino-2-phenylindole (DAPI, Beyotime). At least five random images for each experimental condition were captured using a confocal laser scanning microscope (ZESSI). Mean fluorescence intensity was performed with image J by experimental staff blinded to the experiments (n = 3).

### Immunofluorescence staining of retinal sections

Paraffin sections of eyeballs were deparaffinized with xylene and alcohol series (n = 3). After washing the slices, endogenous peroxidase activity was blocked with 3% hydrogen peroxide (Servicebio) for 5 minutes. Sections were immersed in 0.01 M citrate antigen retrieval solution (pH 6.0) for antigen retrieval and heated in a microwave oven, followed by cooling for 40 minutes. Samples were then incubated with primary antibody overnight at 4°C, followed by incubation with goat anti-rabbit IgG H&L Alexa Fluor 488 (# ab150077; 1:200; abcam) or anti-mouse IgG Alexa Fluor 594 (1:200; Beyotime) for 1 hour at room temperature. DAPI (#G1012; Servicebio, Wuhan, China) staining was performed 5 minutes. Sections were photographed using a fluorescence microscope (Nikon, Tokyo, Japan).

### Hematoxylin-eosin (H&E) staining

Rats were sacrificed 3 days after intravitreal injection. Eyeballs were fixed in 4% formalin, dehydrated in a graded series of ethanol (80%, 95%, 100%) and embedded in paraffin (n = 5). Then the eyeball was cut into 3 µm vertical sections. The slices were stained with hematoxylin and eosin (H&E) and visualized using a light microscope (Nikon; Tokyo, Japan) and analyzed using CaseViewer software (3DHISTEC; Sysmex, Switzerland). The thickness of the retinal ganglion cell body complex (GCC) was measured at 1000, 2000, 3000 and 4000 μm from the optic nerve center.

### Flash visual evoked potential (f-VEP) analysis

The rats were anesthetized with pentobarbital 3 days after drug intravitreal injection (n = 3). After 15 min of dark adaptation, we fixed the and inserted electrodes under the skin of the back (ground electrode), anterior bregma (cathode), and the occipital bone (anode). Covering the contralateral eye with thick gauze and foil, recorded and evaluate the visual function with a multifocal electroretinography recorder (Gotec; GT-2008 V–VI; Chongqing, China) and the Ganzfeld system (Gotec; Chongqing, China).

### Evaluation of anterograde axon transport by cholera toxin β-subunit (CTB)

One day after drug treated, 2 uL CTB-Alexa 555 (#C34776; Thermofisher; Waltham, MA) was intravitreally injected (n = 3). Two days later, the rats were anesthetized. The optic nerves were embedded in OCT (Tissue-Tek; Sakura Finetek Inc; Tokyo, Japan) and sliced into 15 µm sections. Using a fluorescent microscope to get images.

### Literature search strategy

To ensure a comprehensive understanding of the current knowledge landscape, a structured literature search was conducted prior to the initiation of this study. We searched PubMed, Web of Science, and Google Scholar databases for articles published up to March 2025 using combinations of the following keywords: “glaucoma,” “retinal ganglion cells,” “m6A methylation,” “ferroptosis,” “epitranscriptomics,” “Mettl3,” “HMGCS1,” and “glutamate excitotoxicity.” Boolean operators (AND, OR) were used to refine search results. Priority was given to peer-reviewed original research articles and high-quality reviews published in English. Articles were screened based on relevance to the study objectives, recency, and methodological rigor. Additional references were identified through manual searching of the bibliographies of selected articles.

### Statistical analysis

All experiments were conducted using randomized group allocation. The group assignments were concealed from the investigators performing the experiments and data analyses to ensure objectivity. Specifically, different individuals were responsible for group allocation, intervention administration, data collection, and outcome assessment. Image quantification and histological evaluations were conducted under blinded conditions. All data are presented as mean ± standard deviation (SD) from at least three independent experiments. Statistical analyses were conducted using GraphPad Prism Version 8.0 (GraphPad Software, San Diego, CA, USA). For comparisons between two groups, a two-tailed Student’s t-test was employed. For comparisons among multiple groups, one-way analysis of variance (ANOVA) followed by post hoc Tukey’s test was applied. A *P* value <0.05 was considered statistically significant.

## Results

### NMDA-induced elevation of m6A RNA methylation levels in rat retinas

**Figure 1.**
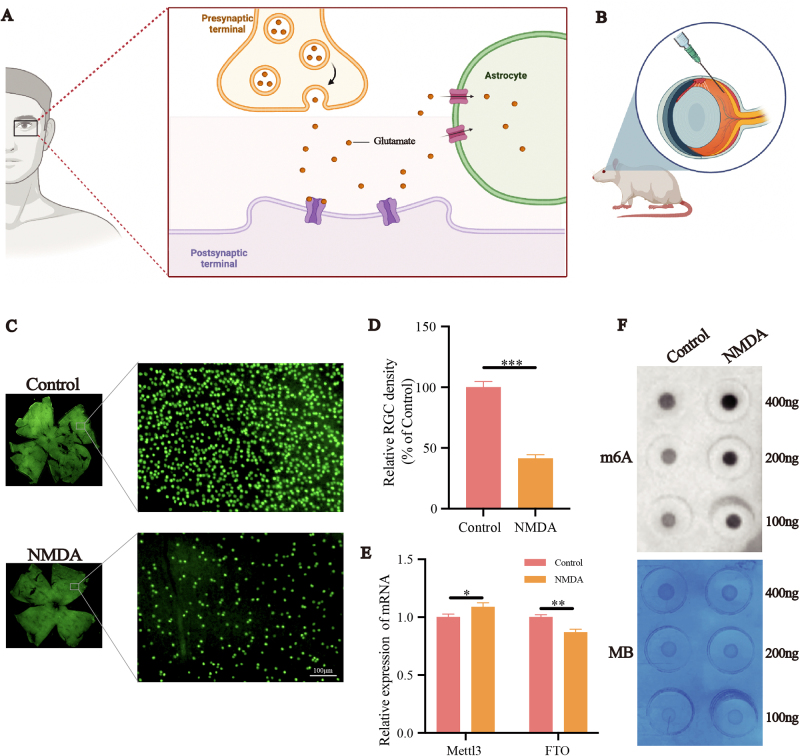
m6A RNA methylation levels and methyltransferase expression changes in glutamate excitotoxicity-induced glaucoma model of rat. (A) A schematic model for the mechanism of NMDA-induced retinal excitotoxic in RGCs. (B) Schematic diagram of constructing NMDA-induced glaucoma model of rat. Qualitative observation (C) and quantitative analysis (D) of the NMDA-induced RGC injury in rat retina (n = 5). Three days after intravitreal injection of NMDA, retinal plating was performed. RGCs were fluorescently labeled with Brn3a antibody and surviving RGCs exhibited strong green fluorescence under a fluorescence microscope. Scale bar: 100 μm. (E) mRNA level of Mettl3 and FTO in retina of NMDA-induced glaucoma model of rat. (n = 3) (F) The RNA methylation level of poly(A) + RNAs isolated from total RNA of the retina of NMDA-induced glaucoma model of rat was indicated by m6A dot blot. Corresponding RNAs were loaded equally by a 2-fold serial dilution with 400 ng, 200 ng and 100 ng. Methylene blue staining served as a loading control. *P < 0.05, **P < 0.01, ***P < 0.001 (one-way analysis of variance followed by Tukey’s multiple comparison test). Data are expressed as the means ± SD. At least three independent experiments were repeated.

Previous studies have indicated that excessive amount of glutamate can lead to overactivation of NMDA receptors in RGCs, inducing excitotoxicity and resulting in RGC death. In line with previous research, we induced glutamate excitotoxicity in rat RGCs by intravitreal injection of NMDA^[37,38]^ (Fig. 1A). Refer to the previous studies,^[39–41]^ we assessed the impact of intravitreal NMDA injection on rat RGCs through retinal flat-mount immunofluorescence staining (Fig. 1B). Compared to the control group, RGCs in the NMDA group were significantly reduced, approximately 40% of the control group (*P* < 0.05) (Fig. 1C-D). Subsequently, we extracted retinal RNA for qPCR experiments. 3 days after the NMDA being injected into the vitreous cavity, the mRNA level of the methylation enzyme Mettl3 in the retina increased to approximately 1.1 times that of the control group (*P* < 0.05), while the mRNA level of the demethylation enzyme FTO decreased to approximately 0.8 times that of the control group (*P* < 0.05) (Fig. 1E). m6A levels are maintained in balance through the coordinated regulation of methyltransferases and demethylases^[42]^. We utilized m6A dot blot to detect changes in m6A RNA modification levels in total retinal RNA and found that, compared to the control group, the NMDA group exhibited darker spot intensities (Fig. 1F). In summary, elevated Mettl3 expression and reduced FTO expression induced by NMDA in the retina lead to increased m6A RNA modification levels, potentially contributing to retinal RGC damage.

### m6A RNA methylation changes in rat ratina after NMDA treatment

**Figure 2.**
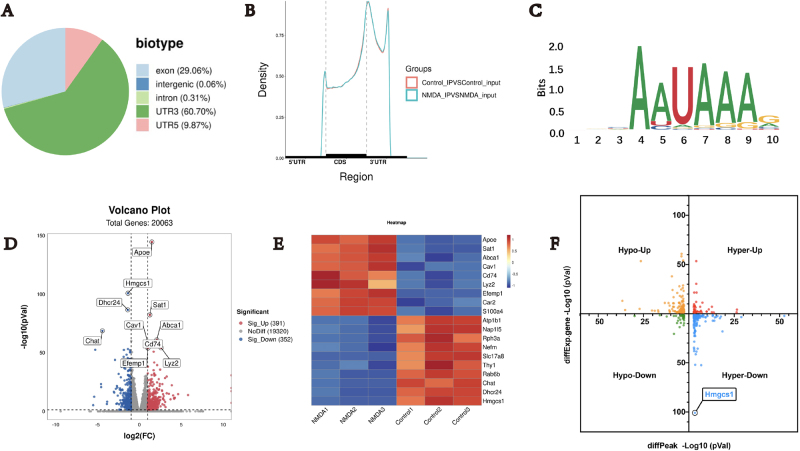
m6A RNA methylation. (A) Distribution of peak on gene functional elements. (B) Peak density in different region. (C) Enrichment for known m6A consensus motif RRACH. (D) Volcano plot of differentially expressed genes between NMDA and control groups. (E) The heat map shows NMDA-induced top 20 genes. (F) m6A-seq and RNA-seq analysis of differentially expressed genes between NMDA and control group.

To obtain the transcriptome-wide m6A RNA methylation map in retina, a series of m6A-immunoprecipitation (IP) and the matched input (non-IP control) libraries was constructed and sequenced. This series included retina tissue of control and NMDA group, with three biological replicates each. Raw sequencing reads were processed to discard adaptor sequences and low-quality bases using the Trimmomatic v0.36 tool (Bolger *et al*). The m6A peaks in retinas are abundant in 3′-UTRs (60.70% of m6A peaks) (Fig. 2A). Enrichment analysis in each segment showed that gene peak mostly enriched in 3′-UTR segment (Fig. 2B). The m6A consensus motif in the retina was identified as “RGACH” by HOMER software based on our m6A-sequence (Fig. 2C). To further investigate the mechanism of the NMDA on the retina, RNA sequencing analysis was performed. Compared to the control group, the NMDA treatment group showed 391 upregulated genes and 352 downregulated genes (Fig. 2D). These genes included lipid metabolism pathway-related genes, such as Apoe and Abca1 (Fig. 2E). These results suggest that NMDA exert their RGC-damage effects by regulating the transcriptomic abnormalities in the retina. In the m6A-seq and RNA-seq analysis, m6A-containing transcripts and different expressed mRNA revealed that the transcript level of HMGCS1 is down, while its m6A RNA methylation level is up, indicating the HMGCS1 is a potential target of RGC protection (Fig. 2F).

### Inhibition of Mettl3 reduces glutamate excitotoxicity-induced ferroptosis in R28 cells

**Figure 3.**
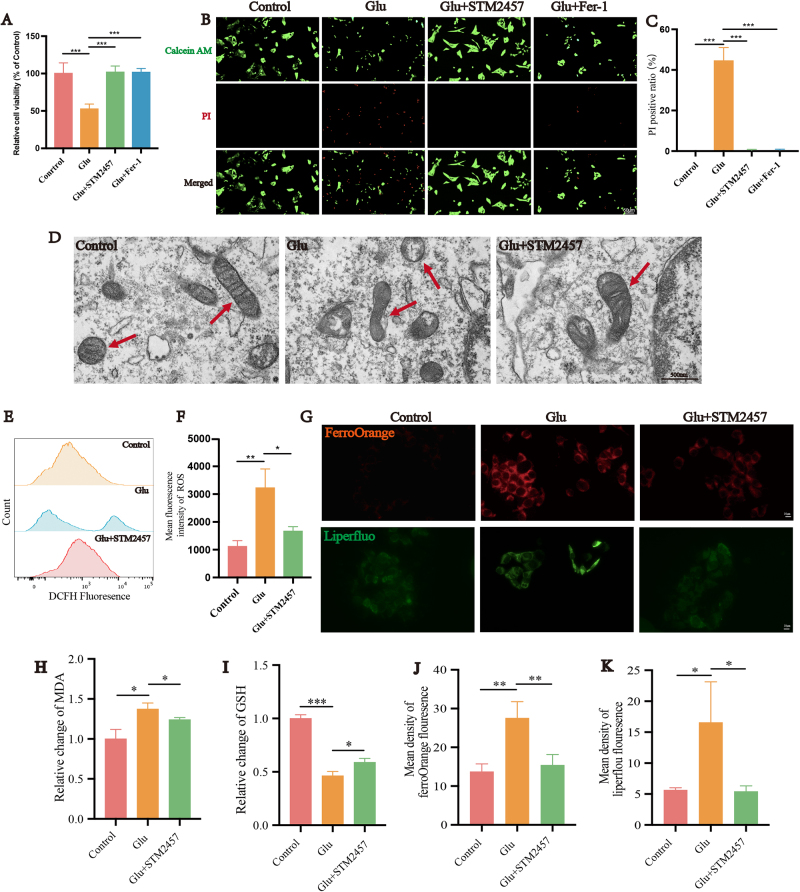
Inhibition of Mettl3 reduce glutamate excitotoxicity-induced ferroptosis in R28 cells. (A) Effect of STM2457 and Fer-1 on the cell viability of R28 cells treated with 10 mM glutamate for 24 hours (n = 3). (B) Propidium iodide (PI; red) and Calcein AM (green) staining of R28 cells treated with glutamate, STM2457 and Fer-1 as indicated for 24 hours. Scale bar: 50 μm. (C) Quantification of the results shown in B (n = 5). (D) Transmission electron microscope images of R28 cells treated with glutamate and STM2457. Scale bar: 500 nm. (E, F) Flow cytometry was used to detect the effect of STM2457 on lipid ROS production in R28 cells treated as indicated for 24 hours (n = 4). (G) R28 cells treated as indicated were stained with the fluorescent probe Mito-FerroOrange to determine ferrous ion levels. FerroOrange undergoes an irreversible reaction with intracellular Fe^2+^ in live cells, resulting in orange fluorescence emission. Ex: 543 nm and Em: 580 nm. Scale bar: 10 μm. R28 cells treated as indicated were stained with Liperfluo for measurement of lipid peroxides. Liperfluo is selectively oxidized by lipid peroxides, and the oxidized form of Liperfluo exhibits strong green fluorescence at the cell membrane. Scale bar: 10 μm. MDA (H) and GSH (I) levels in R28 cells after glutamate and STM2457 treatment (n = 3). (J) Quantification of mean density of FerroOrange fluorescence in the groups (n = 5). (K) Comparison of mean density of Liperfluo fluorescence in the groups (n = 5). *P < 0.05, **P < 0.01, ***P < 0.001 (one-way analysis of variance followed by Tukey’s multiple comparison test). Data are expressed as the means ± SD. At least three independent experiments were performed.

Previous research has confirmed that glutamate serves as a classic inducer of ferroptosis^[43]^. Our previous study confirmed that glutamate excitotoxicity can induce ferroptosis in RGCs within the R28 cell line and the retinas of SD rats^[11]^. In order to investigate the impact of Mettl3-mediated m6A RNA methylation on glutamate excitotoxicity-induced ferroptosis in the R28 cells line, we used the Mettl3 inhibitor STM2457 and conducted CCK-8 assays (Fig. 3A). As expected, the cell viability of the glutamate group decreased to approximately 50% compared with the control group (*P* < 0.05). Consistent with the previous studies, cell viability significantly increased after the application of the ferroptosis inhibitor Fer-1 (*P* < 0.05). The use of the Mettl3 inhibitor STM2457 demonstrated an effect comparable to Fer-1, effectively preventing cell death induced by glutamate (*P* < 0.05). For a more visual observation of the impact of Mettl3 inhibition on R28 cells, we performed Calcein/PI staining (Fig. 3B-C). The results were consistent with the CCK-8 assay. In the control group, live cells were stained green with Calcein, and no cells marked with red fluorescence of PI were detected. The glutamate group exhibited a large number of red-stained dead cells, while the Fer-1 and STM2457 groups showed hardly any red-stained cells. Transmission electron microscopy revealed that the glutamate group showed morphological changes indicative of ferroptosis, such as mitochondrial shrinkage, reduction of mitochondrial cristae and mitochondrial outer membrane rupture. Notably, the mitochondrial damage induced by glutamate was prevented by STM2457 treatment (Fig. 3D). Lipid peroxidation, intracellular iron accumulation, and loss of antioxidant defense are crucial factors in inducing ferroptosis^[20]^. To better understand the role of Mettl3 in ferroptosis, we used flow cytometry to detect intracellular ROS levels (Fig. 3E-F). The average fluorescence intensity (MFI) of ROS in the glutamate group significantly increased to approximately 2.9 times that of the control group (*P* < 0.05). In the STM2457 group, the MFI of ROS decreased to about 1.5 times that of the normal group (*P* < 0.05). Also, other indicators of ferroptosis, such as GSH and MDA levels, were also assessed. We employed Mito-ferroOrange to measure intracellular free iron content (Fig. 3G, J). The fluorescence intensity of FerroOrange in the glutamate group significantly increased to approximately 2 times that of the control group (*P* < 0.05). The STM2457 group reduced to about 1.1 times that of the control group (*P* < 0.05). Then we used Liperfluo to detect intracellular lipid peroxides (Fig. 3G, K). The fluorescence intensity of Liperfluo in the glutamate group increased to about 3 times that of the control group (*P* < 0.05). After adding STM2457, it decreased to about 1 time that of the control group (*P* < 0.05). MDA levels significantly increased to about 1.37 times that of the normal group in the glutamate-treated group, while the STM2457 group exhibited a slight decrease to about 1.24 times that of the control group (*P* < 0.05) (Fig. 3H). GSH levels in the glutamate group decreased to approximately 0.46 times that of the control group (*P* < 0.05). The STM2457 group showed a slight increase in GSH, approximately 0.59 times that of the control group (*P* < 0.05) (Fig. 3I).

### Mettl3 inhibition reduces glutamate excitotoxicity-induced ferroptosis through SLC7A11/GSH/GPX4 pathway

**Figure 4.**
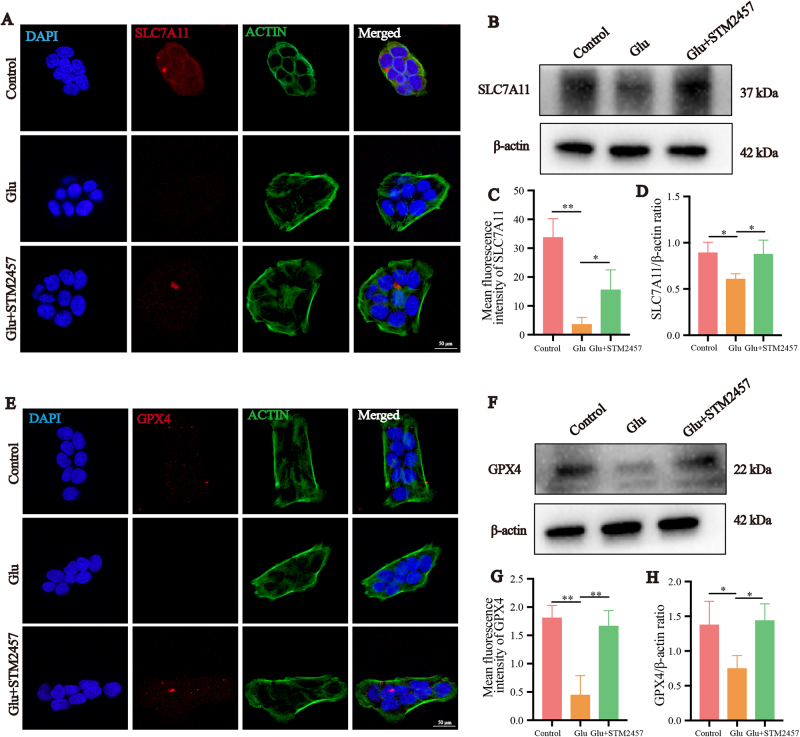
Mettl3 inhibition reduces glutamate excitotoxicity-induced ferroptosis through SLC7A11/GSH/GPX4 pathway. (A) Confocal microscopy was used to detect the intracellular SLC7A11 protein levels. The protein was labeled with red fluorescence, the cytoskeleton was labeled with green fluorescence, and the nucleus was stained with DAPI, showing blue fluorescence. (B) SLC7A11 protein expression in R28 cells with glutamate or STM2457 treated. (C) Quantification analysis of SLC7A11 protein mean fluorescence intensity in R28 cells detected by Confocal microscopy (n = 5). (D) Quantification analysis of SLC7A11 protein expression in R28 cells with glutamate or STM2457 treated detected by western blot (n = 3). (E) Confocal microscopy was used to detect the intracellular GPX4 protein levels. The protein was labeled with red fluorescence, the cytoskeleton was labeled with green fluorescence, and the nucleus was stained with DAPI, showing blue fluorescence. (F) GPX-4 protein expression in R28 cells with glutamate or STM2457 treated. (G) Quantification analysis of GPX4 protein mean fluorescence intensity in R28 cells detected by Confocal microscopy (n = 5). (H) Quantification analysis of GPX4 protein expression in R28 cells with glutamate or STM2457 treated detected by western blot (n = 3). *P < 0.05, **P < 0.01 (one-way analysis of variance followed by Tukey’s multiple comparison test). Data are expressed as the means ± SD. At least three independent experiments were performed.

The SLC7A11/GSH/GPX4 axis represents the principal intracellular antioxidant defense system against ferroptosis. Our previous experiments demonstrated that GSH levels were significantly decreased in the glutamate-treated R28 cells compared to the control group. Notably, treatment with STM2457 partially alleviated the glutamate-induced reduction in GSH (Fig. 3I). To further elucidate the changes in SLC7A11 and GPX4 expression in R28 cells, we performed immunofluorescence and Western blotting analyses. The results showed that the protein expression of SLC7A11 was decreased in the glutamate-treated group compared to the control, whereas treatment with glutamate + STM2457 significantly upregulated SLC7A11 expression compared to glutamate alone (*P* < 0.05) (Fig. 4A-D). Similarly, GPX4 protein expression was reduced in the glutamate-treated group compared to the control, while the glutamate + STM2457 group exhibited a significant increase in GPX4 protein expression relative to the glutamate-only group (*P* < 0.05) (Fig. 4E-H).

### HMGCS1 inhibits ferroptosis in R28 cells via an m6A RNA methylation-dependent mechanism

**Figure 5.**
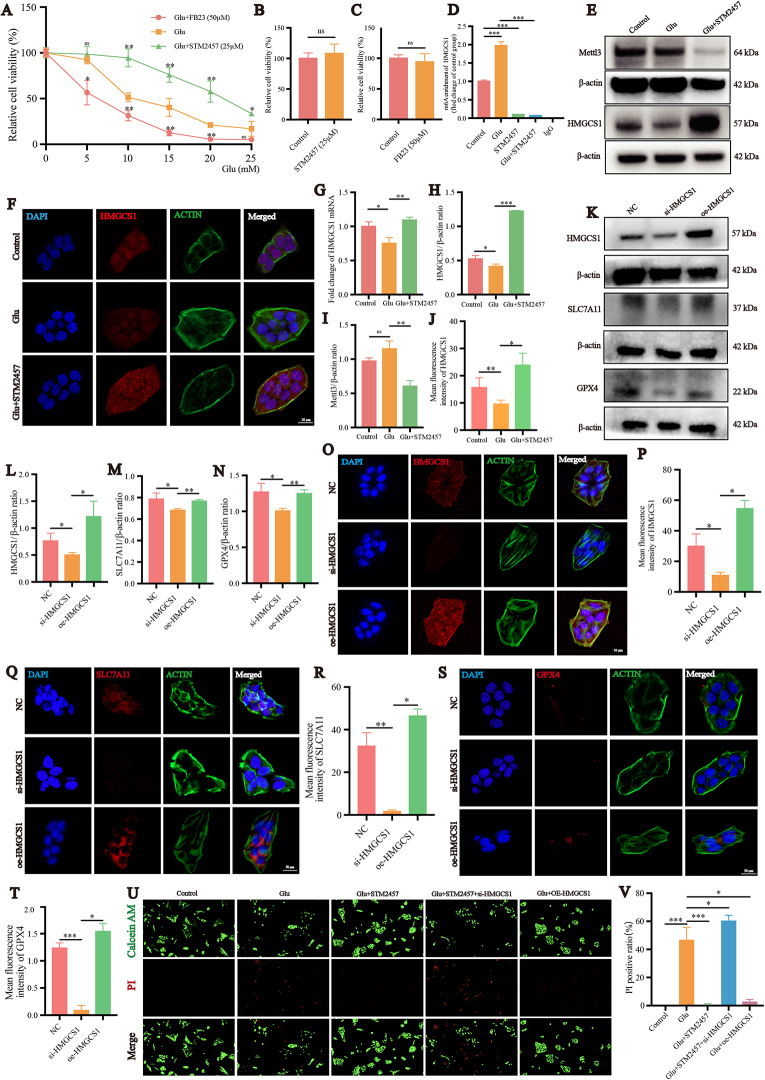
HMGCS1 inhibits ferroptosis in R28 cells in an m6A-dependent manner. (A) Effect of STM2457 (25 μM) and FB23 (50 μM) on the cell viability of R28 cells treated with 5, 10, 15, 20, 25 mM glutamate for 24 hours (n = 3). (B, C) Effect of STM2457 (25 μM) and FB23 (50 μM) on the cell viability of R28 cells (n = 3). (D) MeRIP-qPCR analysis of HMGCS1 after treated with glutamate (10 mM) and STM2457 (25 μM) for 24 hours in R28 cells. (E) Mettl3 and HMGCS1 protein expression in R28 cells treated with glutamate (10 mM) and STM2457 (25 μM) for 24 hours (n = 3). (F) Confocal microscopy was used to detect the intracellular HMGCS1 protein levels. The protein was labeled with red fluorescence, the cytoskeleton was labeled with green fluorescence, and the nucleus was stained with DAPI, showing blue fluorescence. (G) mRNA level of HMGCS1 in R28 cells treated with glutamate (10 mM) and STM2457 (25 μM) for 24 hours (n = 3). (H) Quantification analysis of HMGCS1 protein expression in R28 cells (n = 3). (I) Quantification analysis of Mettl3 protein expression in R28 cells (n = 3). (J) Quantification analysis of HMGCS1 protein mean fluorescence intensity in R28 cells (n = 5). (K) HMGCS1, SLC7A11 and GPX-4 protein expression in R28 cells with HMGCS1 knockdown or overexpress (n = 3). Quantification analysis of HMGCS1 (L), SLC7A11 (M) and GPX4 (N) protein expression in R28 cells (n = 3). (O) Confocal microscopy was used to detect the intracellular HMGCS1 protein levels. (P) Quantification analysis of HMGCS1 protein mean fluorescence intensity in R28 cells (n = 5). (Q) Confocal microscopy was used to detect the intracellular SLC7A11 protein levels. (R) Quantification analysis of SLC7A11 protein mean fluorescence intensity in R28 cells (n = 5). (S) Confocal microscopy was used to detect the intracellular GPX4 protein levels. (T) Quantification analysis of GPX4 protein mean fluorescence intensity in R28 cells (n = 5). (U) PI (red) and Calcein AM (green) staining of R28 cells treated with glutamate, STM2457, si-HMGCS1 or oe-HMGCS1 as indicated for 24 hours. Scale bar: 50 μm. (V) Quantification of the results shown in U (n = 5). *P < 0.05, **P < 0.01, ***P < 0.001, ns: *P* > 0.05 (one-way analysis of variance followed by Tukey’s multiple comparison test). Data are expressed as the means ± SD. At least three independent experiments were repeated.

The Mettl3 inhibitor STM2457 (25 μM) and FTO inhibitor FB23 (50 μM) were used separately on R28 cells in a glutamate model. CCK-8 assay was conducted, revealing that with increasing glutamate concentrations, cell viability in the glutamate group gradually decreased (Fig. 5A). The cell viability in 10 mM glutamate group was around 50% that of the control group (*P* < 0.05). STM2457 to some extent prevented the glutamate-induced excitotoxicity-mediated decrease in cell viability, with cell viability in the STM2457 group at around 100% (*P* < 0.05). FB23 exacerbated glutamate-induced damage, leading to further reduction in cell viability compared to the glutamate group (*P* < 0.05). Using 25 μM STM2457 or 50 μM FB23 alone had no impact on R28 cell viability (*P* > 0.05) (Fig. 5B-C). It further confirmed that the positive correlation between m6A RNA methylation level and ferroptosis in the glutamate R28 cells model. To further investigate the changes in m6A RNA methylation modification levels on HMGCS1 mRNA during this process, MeRIP-qPCR was performed (Fig. 5D). Glutamate treatment significantly increased m6A RNA methylation modification levels on HMGCS1 mRNA by 1.97-fold of the control group (*P* < 0.05). Regardless of glutamate exposure, the use of STM2457 significantly decreased m6A RNA methylation modification levels on HMGCS1 mRNA (*P* < 0.05). qPCR results aligned with sequencing data, HMGCS1 mRNA levels in the glutamate group reduced to 0.75-fold of the control group (*P* < 0.05), which increased to 1.09-fold in the STM2457 group (*P* < 0.05) (Fig. 5G). Based on qPCR results, western blotting was performed to assess the relative expression levels of Mettl3 and HMGCS1 proteins (Fig. 5E, H-I). Although qPCR experiments in in-vivo studies showed a significant increase in Mettl3 mRNA levels in the NMDA group compared to the control group (*P* < 0.05) (Fig. 1E). *In vitro* experiments showed that Mettl3 protein levels appeared higher in the glutamate group compared to the control group, but the difference was not statistically significant (*P* = 0.0647). This lag in protein level changes compared to mRNA level changes might explain this discrepancy. The HMGCS1 protein expression level was reduced in the glutamate group compared to the control group, while the HMGCS1 protein expression level was significantly increased in the STM2457 group compared to the glutamate group (*P* < 0.05). The changes in the trend observed in the results of cellular immunofluorescence are consistent with those of the Western blot results (Fig. 5F, J). Thus, it can be concluded that using STM2457 to inhibit Mettl3 reduces mRNA m6A RNA methylation levels on HMGCS1 and increases protein expression levels in the glutamate model.

To further investigate the role of HMGCS1 in the glutamate induced ferroptosis, we successfully constructed oe-HMGCS1 and si-HMGCS1 to regulate the HMGCS1 expression in R28 cells (Fig. 5K-L) (Supplemental Digital Content, Figure 1A, available at: http://links.lww.com/JS9/E901). The changes in the trend observed in the results of cellular immunofluorescence are consistent with those of the Western blot results (Fig. 5O-P). To observe the effects of oe-HMGCS1 and si-HMGCS1, we conducted Western blotting analyses (Fig. 5K, M-N). The results revealed that SLC7A11 protein expression was decreased in the si-HMGCS1 group compared to the NC group, whereas SLC7A11 expression was significantly upregulated in the oe-HMGCS1 group relative to the si-HMGCS1 group (*P* < 0.05). Similarly, GPX4 protein expression was lower in the si-HMGCS1 group compared to the control, while GPX4 expression was significantly increased in the oe-HMGCS1 group compared to the si-HMGCS1 group (*P* < 0.05). The changes in the trend observed in the results of cellular immunofluorescence are consistent with those of the Western blot results (Fig. 5Q-T). We conducted Calcein/PI staining (Fig. 5U-V). In the control group, a large number of live cells stained with green fluorescence were visible, and there were no dead cells, while a substantial number of dead cells with red staining were observed in the glutamate group. In comparison to the glutamate group, the Glu + STM2457 + si-HMGCS1 group showed an increase in red-stained cells, indicating that knocking down HMGCS1 exacerbated the damage induced by glutamate. While red-stained cells percentage decreased in Glu + oe-HMGCS1 group compared to glutamate group.

### YTHDF2 specifically recognizes the m6A-methylated sites on HMGCS1 mRNA, mediating the degradation of HMGCS1 RNA

**Figure 6.**
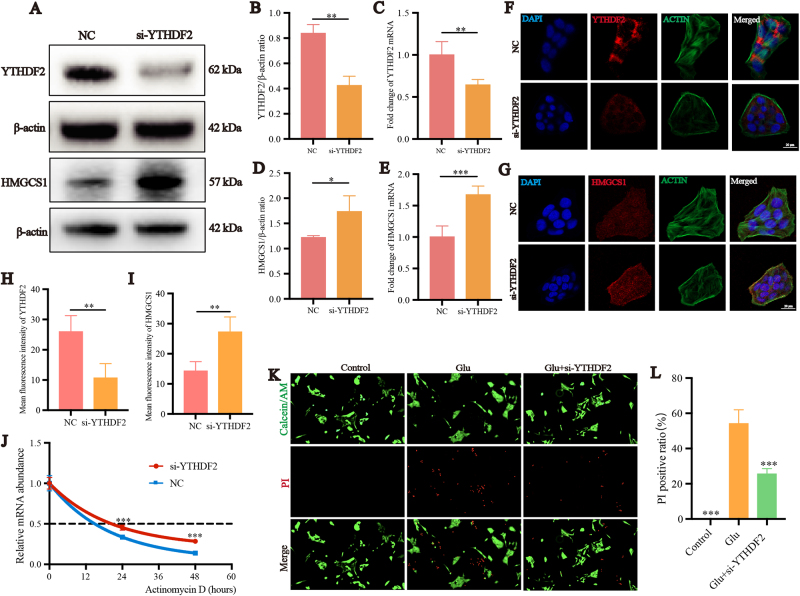
YTHDF2 specifically recognizes the m6A-methylated sites on HMGCS1 mRNA, mediating the degradation of HMGCS1 RNA. (A–C) YTHDF2 mRNA and protein expression in R28 cells with YTHDF2 knockdown. (D, E) HMGCS1 mRNA and protein expression in R28 cells with YTHDF2 knockdown. Confocal microscopy was used to detect the intracellular YTHDF2 (F) and HMGCS1 (G) protein levels. The protein was labeled with red fluorescence, the cytoskeleton was labeled with green fluorescence, and the nucleus was stained with DAPI, showing blue fluorescence. (H, I) Quantification analysis of YTHDF2 and HMGCS1 protein mean fluorescence intensity in R28 cells (n = 5). (J) HMGCS1 mRNA decay assay using actinomycin D treatment in R28 cells transfected with si-YTHDF2 (n = 3). (K) PI (red) and Calcein-AM (green) staining of R28 cells treated with glutamate, STM2457 and si-HMGCS1 as indicated for 24 hours. Scale bar: 50 μm. (L) Quantification of the results shown in K (n = 5). *P < 0.05, **P < 0.01, ***P < 0.001 (one-way analysis of variance followed by Tukey’s multiple comparison test). Data are expressed as the means ± SD. At least three independent experiments were repeated.

Due to the reduction of m6A RNA methylation, there seems to be an increase in the expression of HMGCS1. We hypothesize that HMGCS1 mRNA is a target of YTHDF2, leading to increased degradation of m6A-modified HMGCS1 mRNA. We constructed si-YTHDF2 and successfully lowered the mRNA and protein expression levels of YTHDF2 (Fig. 6A-C). Upon YTHDF2 knockdown, we observed elevated levels of HMGCS1 mRNA and protein expression in R28 cells compared to the control group (*P* < 0.05) (Fig. 6A, D-E). The changes in the trend observed in the results of cellular immunofluorescence are consistent with those of the Western blot results (Fig. 6F-I). We conducted mRNA decay assays in YTHDF2 knockdown (KD) cells (Fig. 6J). R28 cells were stably cultured and then incubated with actinomycin D for 0, 24, or 48 hours before RNA extraction. Quantitative RT-PCR analysis was used to determine the half-life of HMGCS1 mRNA. Compared to control cells, the half-life of HMGCS1 mRNA was significantly prolonged in YTHDF2 KD cells, indicating that m6A modification is involved in the regulation of HMGCS1 mRNA stability. To observe the effect of si-YTHDF2, we performed Calcein AM/PI staining (Fig. 6K). In the control group, numerous live cells stained green with Calcein were visible. The glutamate group displayed a significant number of red fluorescence dead cells. In contrast to the glutamate group, the si-YTHDF2 group showed fewer dead cells (Fig. 6L). This suggests that the decreased degradation of HMGCS1 mRNA following YTHDF2 knockdown leads to a relative increase in HMGCS1 protein, mitigating excitotoxicity-induced ferroptosis in R28 cells to some extent.

### Inhibition of Mettl3 protects RGCs in the NMDA-induced glaucoma model of rats by regulating m6A RNA methylation of HMGCS1

**Figure 7.**
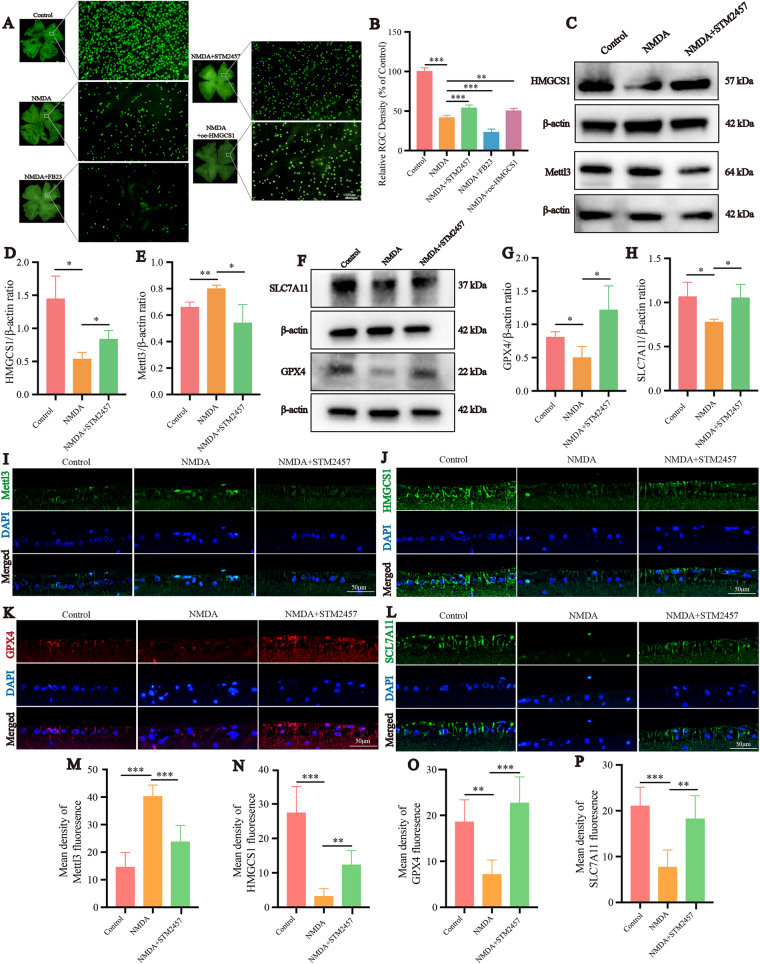
STM2457 protects RGCs in the NMDA-induced glaucoma model of rat by regulating HMGCS1. Qualitative observation (A) and quantitative analysis (B) of the STM2457, FB23 and oe-HMGCS1 effect on NMDA-induced RGC injury in rat retina (n = 5). Three days after intravitreal injection of NMDA, retinal plating was performed. RGCs were fluorescently labeled with Brn3a antibody and surviving RGC exhibited strong green fluorescence under a fluorescence microscope. Scale bar: 100 μm. (C–E) HMGCS1 and Mettl3 protein expression in R28 cells with NMDA and STM2457 treated. (F–H) SLC7A11 and GPX-4 protein expression in R28 cells with NMDA and STM2457 treated. Three days after intravitreal injection of NMDA and STM2457, paraffin sections were collected and processed for immunofluorescence experiments to measure the fluorescence intensity of Mettl3 (I, M), HMGCS1 (J, N), GPX4 (K, O), and SLC7A11 (L, P) of the retinal sections in each group under a fluorescence microscope (n = 3). Scale bars: 50 μm. *P < 0.05, **P < 0.01, ***P < 0.001 (one-way analysis of variance followed by Tukey’s multiple comparison test). Data are expressed as the means ± SD. At least three independent experiments were repeated.

To further investigate the role of Mettl3 in glutamate excitotoxicity in rat models, intravitreal injections of NMDA, STM2457, FB23, or oe-HMGCS1 were performed, followed by retinal flat-mount immunofluorescence assays (Fig. 7A-B). As expected, compared to the NMDA group, the NMDA + STM2457 group and NMDA + oe-HMGCS1 group exhibited increased RGCs, while the NMDA + FB23 group showed a decrease (*P* < 0.05). TUNEL staining on retinal paraffin sections was used to observe apoptotic cells in the retina (Supplemental Digital Content, Figure 1D, available at: http://links.lww.com/JS9/E901). Green-stained apoptotic cells were visible in the RGC layer of the NMDA group’s retina, while no green fluorescence was observed in the control group or the NMDA + STM2457 group. To confirm the *in vivo* regulation of Mettl3 on HMGCS1, we conducted Western blotting assay, the results showed that HMGCS1 protein expression was decreased in the NMDA group compared to the control group, whereas HMGCS1 expression was significantly increased in the NMDA + STM2457 group compared to the NMDA group (*P* < 0.05). Additionally, Mettl3 protein expression was elevated in the NMDA group compared to the control, while Mettl3 expression was significantly reduced in the NMDA + STM2457 group compared to the NMDA group (*P* < 0.05) (Fig. 7C-E). The expression of YTHDF2 showed no significant differences among the different groups (Supplemental Digital Content, Figure 1B-C, available at: http://links.lww.com/JS9/E901). Also we conducted paraffin section immunofluorescence assays (Fig. 7I-J, M-N). It was found that in the glutamate group, Mettl3 fluorescence intensity was higher in the NMDA group compared to the control group, while in the STM2457 group, Mettl3 fluorescence intensity was reduced compared to the NDMA group. This indicates that STM2457 also exerts an inhibitory effect on Mettl3 *in vivo*. Subsequently, we discovered that the fluorescence intensity of HMGCS1 was reduced in the NMDA group compared to the control group, while in the STM2457 group, the fluorescence intensity of HMGCS1 was enhanced compared to the NMDA group. This is consistent with the results from *in vitro* experiments, inhibiting Mettl3 led to increased HMGCS1 expression, resulting in reduced RGCs loss caused by NMDA. To further investigate the changes in SLC7A11 and GPX4 expression *in vivo*, we performed Western blotting assays. The results showed that protein expression levels of SLC7A11 and GPX4 were reduced in the NMDA-treated group compared to the control group, while treatment with NMDA + STM2457 significantly increased the expression of both proteins compared to the NMDA group (*P* < 0.05) (Fig. 7F-H). The changes in the trend observed in the results of retinal flat-mount immunofluorescence assays are consistent with those of the Western blot results (Fig. 7K-L, O-P).

### Inhibition of Mettl3 improves rat visual functions after NMDA treatment

**Figure 8.**
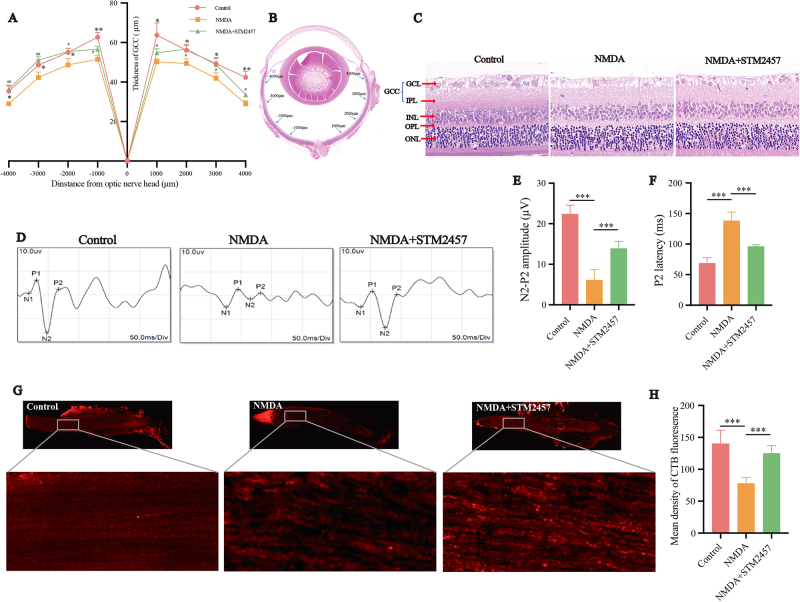
STM2457 improves rat visual function. (A, B) Thickness of the GCC at 1000, 2000, 3000 and 4000 μm from the optic disc 3 days after intravitreal injection of NMDA and STM2457. (C) Effects of STM2457 on retinal morphology in NMDA-induced glaucoma model of rat. H&E staining was performed 3 days after intravitreal injection of NMDA and STM2457. Scale bar: 50 μm. (D–F) Flash visual evoked potentials of rats 3 days after intravitreal injection of NMDA and STM2457 (n = 5). (G, H) One day after intravitreal injection of NMDA and STM2457, CTB-594 was injected. Two days later, paraffin sections were collected and processed for immunofluorescence experiments to measure the fluorescence intensity of CTB-594 (red) of the retinal sections in each group under a fluorescence microscope (n = 3). GCL: Ganglion cell layer; INL: inner nuclear layer; IPL: inner plexiform layer; ONL: outer nuclear layer; RGC: retinal ganglion cell. Inner retina consists of GCL and IPL. *P < 0.05, **P < 0.01, ***P < 0.001 (one-way analysis of variance followed by Tukey’s multiple comparison test). Data are expressed as the means ± SD. At least three independent experiments were performed.

To investigate the impact of Mettl3 inhibition on visual functions in the NMDA-induced glaucoma rat model, we performed H&E staining to observe retinal morphological changes and measured the thickness of the GCC at distances of 1000, 2000, 3000, and 4000 μm from the optic disc (Fig. 8A-C). In the NMDA group, the GCC layer exhibited thinning compared to the control group (*P* < 0.05), while the STM2457 group showed an increase in GCC thickness compared to the NMDA group (*P* < 0.05). f-VEP is a common clinical examination for assessing visual conduction function. We assessed the effects of STM2457 on the latency and amplitude of the P2 wave in the NMDA-induced glaucoma rat model by detecting f-VEP (Fig. 8D). Three days after NMDA treatment, the latency of the P2 wave increased, and the amplitude decreased compared to the control group, indicating retinal dysfunction caused by intravitreal NMDA injection in rats. However, STM2457 treatment partially restored the P2 wave (Fig. 8E-F). To investigate whether Mettl3 inhibition could protect RGCs axons, we injected Alexa Fluor 594-conjugated CTB into the vitreous to retrogradely trace RGCs axons (Fig. 8G-H). After three days of NMDA injection, RGCs axons suffered severe damage. In contrast, following STM2457 treatment, most axons were spared from excitotoxicity, and CTB labeling intensity was restored. Our findings suggest that inhibiting Mettl3 not only protects RGCs bodies but also effectively maintains RGCs axons in the optic nerve, an essential condition for preserving functional vision.

## Discussion

The pathogenesis of glaucoma is extremely complex, involving multiple forms of cell death. Our previous work has revealed that these include apoptosis^[7]^, pyroptosis^[7,8]^, necroptosis^[9]^, ferroptosis^[10,11]^, and PANoptosis^[12]^. These distinct modes of cell death are intricately interconnected, forming a complex regulatory network. However, the underlying mechanisms and common contributing factors remain incompletely understood.

Accumulation of glutamate in the retina is a common feature across different types of glaucoma^[44]^. As a classical inducer of ferroptosis, glutamate can induce ferroptosis in various cell types^[18]^. The system XC^−^ is composed of s SLC7A11 and SLC3A2. It transports cystine into the cell, which is subsequently reduced to cysteine and utilized for intracellular GSH synthesis. This process inhibits GPX4 activity, leading to the accumulation of lipid peroxides and ultimately inducing ferroptosis. Accumulation of glutamate can inhibit system XC^−^, thereby triggering ferroptosis^[43]^. Our recent studies have found that retinal ischemia-reperfusion in acute high IOP glaucoma models leads to ferroptosis of RGCs^[10]^. Moreover, proteomic analyses have revealed that ferroptosis plays a crucial role in RGCs loss in NMDA induced-glaucoma models^[45]^. During ferroptosis, cellular lipid peroxide metabolism is disrupted, and excessive iron accumulation leads to metabolic abnormalities within the cell. This ultimately results in the failure of the antioxidant system, accumulation of lipid peroxides, and induction of cell death. Therefore, we hypothesize that ferroptosis is one of the key mechanisms underlying RGC death in glaucoma and our research indicates that glutamate excitotoxicity can induce ferroptosis in RGCs.

m6A RNA methylation is one of the most prevalent epigenetic modifications in eukaryotic RNA. It plays a critical role by modulating the methylation levels of key factors within the ferroptosis pathway, thereby regulating their expression and controlling the process of cellular ferroptosis. This regulatory mechanism has been implicated in a wide range of diseases, including lung adenocarcinoma^[46]^, bladder cancer^[47]^, diabetic osteoporosis^[47]^, sepsis-associated acute lung injury^[48]^, chronic obstructive pulmonary disease^[49]^, etc. Its specific functional implication in RGCs of glaucoma had not been previously elucidated. Recent studies suggest m6A RNA methylation is closely related to the onset and progression of glaucoma^[50]^. Studies have shown that high IOP or glutamate excitotoxicity may affect m6A modifications, altering gene expression in RGCs, leading to cell damage and death. In glaucoma models, the changes of m6A modification levels, which subsequently affect RGC survival^[50,51]^. m6A RNA methylation regulates multiple signaling pathways involved in the pathogenesis of glaucoma. A recent study demonstrated that regulation of m6A methylation in the mouse retina attenuates ischemia-reperfusion injury^[52]^. However, the absence of transcriptome-wide m6A sequencing in that study limits its ability to elucidate the downstream regulatory mechanisms involved. Further investigation is needed to explore these complex regulatory networks. Our research found a significant increase in m6A RNA methylation during NMDA-induced glutamate excitotoxicity in RGCs, and inhibition of Mettl3 significantly mitigated NMDA-induced RGC death and restored partial visual function in rats. Consequently, we hypothesize that under the pathological conditions of glaucoma, the levels of m6A RNA methylation in the retina may serve not only as a critical indicator for further exploration of RGCs death but also as a potential diagnostic biomarker for glaucoma. This insight holds promise for advancing early diagnosis and treatment strategies for the disease.

We conducted m6A sequencing on NMDA-induced retinal RNA and observed a significant increase in the m6A RNA methylation levels of HMGCS1, a gene associated with the cholesterol synthesis pathway. Additionally, we found a substantial decrease in HMGCS1 expression due to YTHDF2-mediated its RNA decay. HMGCS1 is one of the key enzymes in the cholesterol synthesis pathway and has recently been found to be closely related to ferroptosis. HMGCS1 plays a crucial role in ferroptosis by regulating cholesterol synthesis and lipid metabolism. Studies have shown that HMGCS1 expression levels can affect intracellular GSH levels, an important antioxidant that can inhibit ferroptosis^[29,30]^. SLC7A11, also known as XCT, is a cystine/glutamate transporter^[53]^. SLC7A11 transports cystine intracellularly and oxidizes it to cysteine to catalyze the synthesis of GSH. GSH is an essential factor for GPx4 to scavenge lipid reactive oxygen species^[54,55]^. SLC7A11 reduction can induce ferroptosis by affecting GPx4 activity. Our study found that HMGCS1 can inhibit ferroptosis by modulating the SLC7A11/GSH/GPX4 axis. In addition, previous studies have shown that HMGCS1 can also influence ferroptosis sensitivity by regulating intermediates in the cholesterol synthesis pathway, such as farnesyl pyrophosphate (FPP) and squalene^[56,57]^. Activation of HMGCS1 could promote nasopharyngeal carcinoma ferroptosis resistance^[58]^. Inhibition of HMGCS1 promotes ferroptosis in lung cancer cells^[59]^. HMGCS1 has also been found to be significantly associated with the pathogenesis of glaucoma. Research indicates that HMGCS1 expression levels in RGCs are closely related to cell survival and function^[60]^. By regulating the cholesterol synthesis pathway, HMGCS1 can affect membrane integrity and fluidity, influencing RGC survival and function^[61,62]^. Furthermore, HMGCS1 is a rate-limiting enzyme in sterol synthesis, playing a crucial role in the regulation of intermediate products and metabolites within the cholesterol synthesis pathway. These intermediates, such as squalene^[57,63]^, lanosterol^[64]^, 24S-hydroxycholesterol^[65]^, not only contribute to cholesterol production but also affect antioxidant capacity and resistance to oxidative stress, which is crucial for RGCs protection in glaucoma. Recent studies have observed that cholesterol metabolism is disrupted in glaucoma patients, suggesting a potential role for HMGCS1 that warrants further investigation^[66,67]^. In summary, HMGCS1 may represent a potential therapeutic target for glaucoma. Learning points from this study include the identification of HMGCS1 as a novel downstream target of Mettl3-m6A signaling in ferroptosis, the neuroprotective potential of STM2457 in glaucoma, and the value of integrating epitranscriptomic profiling in ophthalmic disease models.

Despite the promising findings, this study has several limitations that should be acknowledged. First, the pathogenesis of glaucoma is highly complex, involving an interplay of multiple mechanisms and gene interactions. m6A RNA methylation modification can regulate diverse signaling pathways and various forms of cell death^[24,68]^. Our study specifically focused on how m6A RNA methylation regulates ferroptosis in RGCs. As a post-transcriptional epigenetic modification, the regulatory network of m6A RNA methylation is complex and diverse, presenting potential for further investigation. Second, although R28 cells are widely used as a substitute for primary RGCs in glaucoma related research, they do not fully recapitulate the physiological and molecular characteristics of primary RGCs. Third, our *in vivo* experiments employed an NMDA-induced glaucoma model. This acute system may not fully recapitulate the chronic, multifactorial nature of human glaucoma, limiting direct translational relevance. Furthermore, Mettl3 inhibitors also hold promise as potential candidates for clinical translation. However, challenges related to their pharmacokinetics, off-target effects, delivery efficiency, safety profile, and the potential risks associated with intravitreal injection remain to be addressed. In this context, the development of nanotechnology-based, non-invasive delivery systems targeting RGCs offers a strategic direction for our future translational research efforts.

## Conclusion

Our findings reveal that Mettl3-mediated m6A modification of HMGCS1 mRNA can be recognized by YTHDF2, promoting its decay and leading to ferroptosis in R28 cells and RGCs under glutamate-induced cytotoxicity. Inhibiting Mettl3 suppresses ferroptosis by regulating the HMGCS1/SLC7A11/GSH/GPX4 pathway, representing a potential regulatory pathway for glaucoma. Our results provide a potential target for neuroprotection research related to ferroptosis in glaucoma and explore the potential of m6A RNA methylation modification as a diagnostic marker for glaucoma.

## Data Availability

The data generated and/or analyzed during the current study are available upon reasonable request.

## Research registration unique identifying number (UIN)

This study does not involve human subjects.
